# Presynaptic Proteins and Their Roles in Visual Processing by the Retina

**DOI:** 10.1146/annurev-vision-101322-111204

**Published:** 2024-09-02

**Authors:** Wallace B. Thoreson, David Zenisek

**Affiliations:** 1 Departments of Ophthalmology & Visual Sciences and Pharmacology & Experimental Neuroscience, Truhlsen Eye Institute, University of Nebraska Medical Center, Omaha, Nebraska, USA;; 2 Departments of Cellular and Molecular Physiology, Ophthalmology and Visual Sciences, and Neuroscience, Yale University, New Haven, Connecticut, USA;

**Keywords:** synaptic proteins, retinal ribbon synapse, bipolar cells, amacrine cells, horizontal cells, photoreceptor cells

## Abstract

The sense of vision begins in the retina, where light is detected and processed through a complex series of synaptic connections into meaningful information relayed to the brain via retinal ganglion cells. Light responses begin as tonic and graded signals in photoreceptors, later emerging from the retina as a series of spikes from ganglion cells. Processing by the retina extracts critical features of the visual world, including spatial frequency, temporal frequency, motion direction, color, contrast, and luminance. To achieve this, the retina has evolved specialized and unique synapse types. These include the ribbon synapses of photoreceptors and bipolar cells, the dendritic synapses of amacrine and horizontal cells, and unconventional synaptic feedback from horizontal cells to photoreceptors. We review these unique synapses in the retina with a focus on the presynaptic molecules and physiological properties that shape their capabilities.

## 1. INTRODUCTION

The key functions of the retina are to transduce light into neural signals and to then begin extracting key features of the visual world, which are encoded in a train of action potentials sent through the optic nerve to higher visual centers. At the very first synapse in the retina, visual signals collected by photoreceptor cells are conveyed to a dozen or more different bipolar cell (BPC) types. From there, visual information is transmitted to dozens of different types of amacrine cells (ACs) and ganglion cells.

Synapses in the retina have evolved specializations to facilitate their roles in feature extraction. These include pre- and postsynaptic specializations in both molecular composition and structural arrangements (for a review, see Wichmann & Kuner 2022). In this review, we describe some of the presynaptic specializations that regulate neurotransmitter release in rods, cones, BPCs, horizontal cells (HCs), and ACs in the retina and contribute to their neural computations.

**Figure 1:**
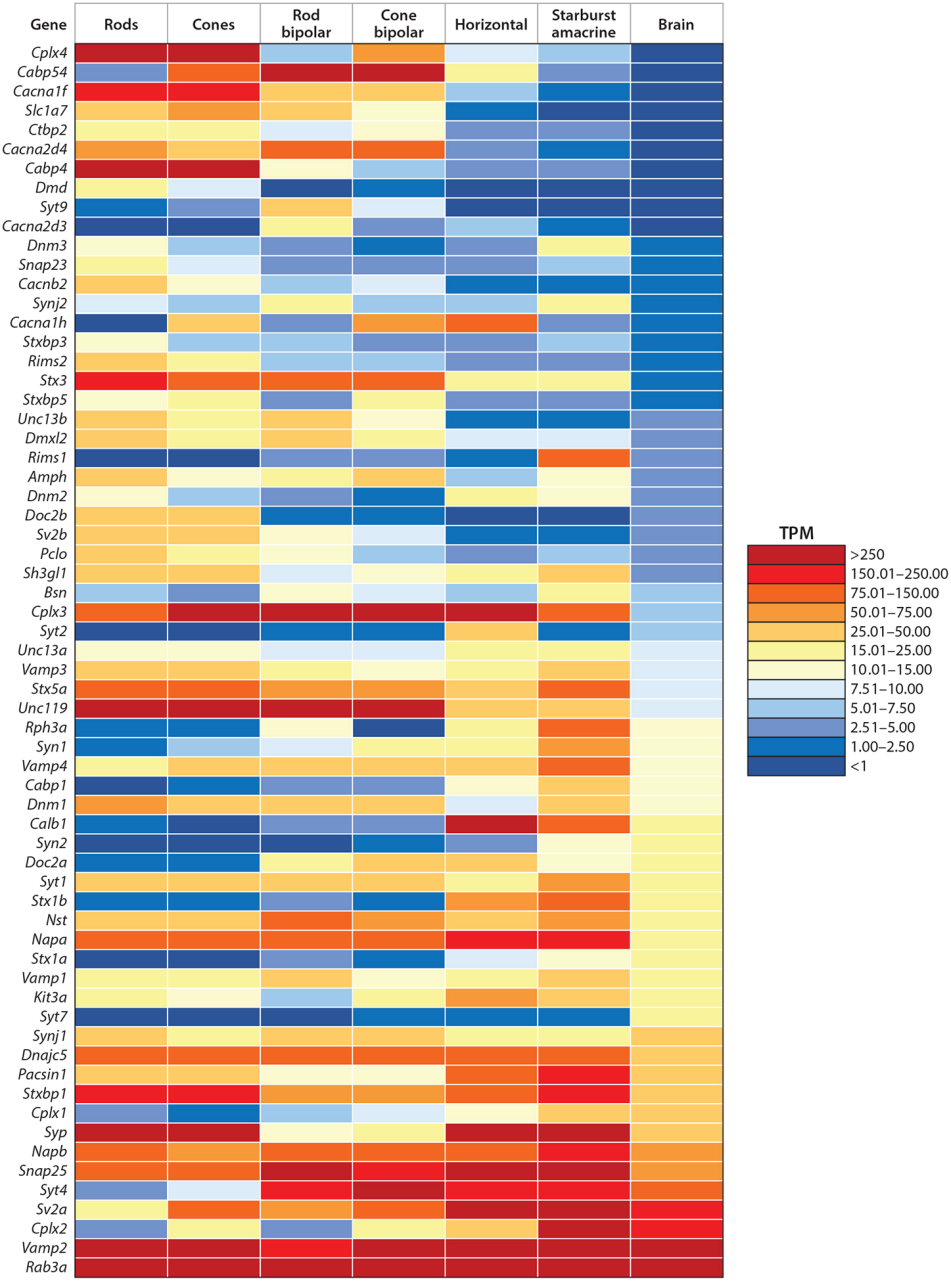
Heat map comparing transcript levels of synaptic proteins from the whole brain to proteins from rods; cones; and rod bipolar, cone bipolar, horizontal, and amacrine cells. Transcripts are arranged in ascending order of expression level in the brain [transcripts per kilobase million (TPM)]. Data for rods, cones, horizontal cells, and starburst amacrine cells taken from Hartl et al. (2017). Data for rod and cone bipolar cells taken from Sarin et al. (2018). Data for the brain taken from Kim et al. (2023).

There is wide heterogeneity in the expression of synaptic proteins both within the retina and when comparing the retina to the brain. Figure 1 illustrates some of these differences with a heat map comparing transcripts for presynaptic proteins in the brain to those expressed in major cell types in the retina: rods; cones; rod BPCs; cone BPCs; HCs; and a single type of AC, the starburst AC (SAC). Transcripts are arranged in ascending order of their expression in the whole brain. Several transcripts show much higher levels in the retina compared to the brain and vice versa. Some of these differences in expression are explained by the presence of a ribbon—a proteinaceous structure that tethers a dense array of vesicles near release sites at graded synapses that are not found in the brain. There are also differences in expression between cell types within the retina: between BPCs and photoreceptor cells, which contain ribbon synapses, and between HCs and ACs, which contain nonribbon synapses.

Transcripts that are especially high in retinal neurons include the Ca^2^+ channel proteins CACNA1F, CACNA2D4, and CACNA2D3 and the Ca^2^+-binding proteins CABP4 and CABP5. As we discuss below, these proteins exhibit unusual features that make them especially suitable for continual regulation of synaptic activity at tonic ribbon synapses. Complexin 4, an isoform of the synaptic protein complexin that facilitates vesicle fusion, is expressed almost exclusively in the retina, while levels of the dominant brain isoforms of complexin, Complexin 1 and 2, are quite low in rods and rod BPCs. Levels of CTBP2, which encodes for both the ribbon protein Ribeye and a more ubiquitous short transcript, are much higher in the retina than the brain (Schmitz et al. 2000). Levels of the SNARE protein syntaxin (STX) 3 are also unusually high in ribbon-bearing cells, with the STX3B splice variant almost exclusively expressed in the retina (Curtis et al. 2008). Table 1 provides an overview of these and other key presynaptic proteins, their functions, and their distribution among retinal cell types. Below, we describe how cell-specific expression of these and other presynaptic proteins may contribute to the unique functions of different retinal synapses.

## 2. RIBBON SYNAPSES OF RODS, CONES, AND BIPOLAR CELLS

Rods, cones, and BPCs have graded synapses, which means that light-dependent changes in membrane potential are encoded by changes in the ongoing rate of vesicle release. These cells possess specialized ribbon-style synapses that are important for normal properties of graded release (Moser et al. 2020, Thoreson 2021). Ribbon synapses are also found in other sensory neurons, including cochlear and vestibular hair cells, pineal photoreceptors, the lateral line organ of fish, and electroreceptors. The structure and function of ribbon synapses in the retina have been the subjects of several recent reviews (Frederick & Zenisek 2023, Moser et al. 2020, Thoreson 2021). In this section, we summarize key features of and highlight some of the remaining questions about ribbon synapses and their role in vision.

### 2.1. Ribbon Structure

In mammals, synaptic terminals of rod photoreceptor cells typically have a single planar ribbon that extends into the cytoplasm, sitting atop a ridge formed by membrane invagination into the terminal. Rod ribbons can be >1 μm in length, wrapping around the synaptic invagination to form a horseshoe shape. Within the synaptic invagination, two rod BPC dendrites terminate beneath the ribbon, with dendrites from two HCs flanking the synaptic ridge, adjacent to the ribbon base (Sterling & Matthews 2005).

Each cone can possess 10–40 ribbons. Cone ribbons are smaller than rod ribbons but also sit above a ridge in the synaptic invagination, with a pair of HC dendrites flanking the ridge and two ON-type BPC dendrites terminating below the ribbon. OFF-type BPCs generally contact the pedicle just outside the invagination. Thus, unlike in most conventional synapses, where a single terminal contacts a single postsynaptic neuron, glutamate released from a single vesicle at a rod or cone ribbon can reach multiple HC and BPC dendrites.

The axon of a BPC terminates in multiple terminals, and each terminal has multiple ribbons; there are thus up to 50 ribbons per cell. Bipolar ribbon synapses lack the deep invaginations of rods and cones, and glutamate released at a single ribbon typically reaches only a pair of postsynaptic AC and/or ganglion cell processes (Sterling & Matthews 2005).

The center of each ribbon is formed from a protein known as Ribeye (Schmitz et al. 2000). Ribeye is a transcript variant of a more ubiquitous protein, CTBP2. CTBP2 is a transcriptional corepressor with homology to the d-isomer-specific 2-hydroxy acid family of dehydrogenases and retains enzymatic activity, albeit with a slow turnover rate (Thio et al. 2004, Turner & Crossley 2001). Ribeye shares this enzymatic domain with the short form of CTBP2 but has an additional ribbon-specific A domain. The role of this enzymatic activity in ribbon function, if any, remains unknown. The unstructured A domains of adjacent Ribeye molecules can interact with one another, self-associating to form dense bodies reminiscent of spherical hair cell ribbons both in culture (Magupalli et al. 2008) and in animals (Chen et al. 2018). Notably, Ribeye in hair cell ribbons is able to move within the ribbon (Graydon et al. 2017) and slowly exchange with a cytoplasmic pool (Chen et al. 2018). Interacting A domains are thought to form a scaffold at the core of the ribbon. The emergence of the flat, planar structures of retinal ribbons involves a ribbon-specific Piccolo splice variant, Piccolino (Michanski et al. 2023, Muller et al. 2019).

Interestingly, the A-domain of Ribeye dates back in evolution to hagfish, *Eptatretus burgeri*, which are chordates but lack vertebrae. Hagfish photoreceptors have spherical synaptic bodies, rather than the platelike ribbons seen in the retinas of true vertebrates (Holmberg & Ohman 1976). BLAST alignment of the Ribeye A domain with the tunicate *Ciona intestinalis*, which lacks ribbons, did not show any significant hits. This suggests that Ribeye and synaptic ribbons evolved after these species diverged. Unlike other vertebrates that we examined (*n* = 10), including the most primitive vertebrate (lamprey), hagfish are unique in lacking the necessary sequences for Piccolo to interact with Ribeye. Although these results are correlative, they are consistent with a role for Piccolino–Ribeye interactions in driving ribbon structure.

Reduction or removal of Ribeye causes the disruption or loss of synaptic ribbons in photoreceptors, BPCs, and hair cells in mice and fish (Becker et al. 2018, Jean et al. 2018, Lv et al. 2016, Maxeiner et al. 2016, Wan et al. 2005). Given the long-standing view that ribbons acted as a reservoir of vesicles needed to resupply release sites in tonic nonspiking neurons, the removal of ribbons was expected to result in a dramatic reduction in synaptic transmission and changes in release kinetics, especially under conditions where release is maximized. The results from ribbonless synapses have proven to be more varied and surprising. Results from photoreceptors indicate that release drops by approximately half, and that vesicle replenishment is slowed, in the absence of ribbons, consistent with an important role for ribbons in continuous release (Mesnard et al. 2022a). In rod BPCs, Ribeye removal was accompanied by a dramatic reduction in total neurotransmitter release but no apparent change in release kinetics. Downstream of the ribbon synapses of the photoreceptors and BPCs, spiking from ON-α ganglion cells in ribbonless mice exhibited normal responses to light flashes but a decreased ability to track high-frequency light stimuli and a decrease in contrast sensitivity (Okawa et al. 2019). By contrast, ribbonless hair cells appear to have essentially normal maximal rates and normal amounts of tonic release but changes in spike timing precision (Becker et al. 2018, Jean et al. 2018, Lv et al. 2016). The different effects of ribbon loss may reflect structural or molecular differences in the types of ribbon synapses in the visual, auditory, and vestibular systems.

### 2.2. Vesicle Mobilization

Conventional synapses in the brain possess multiple pools of vesicles: a synchronous, rapidly releasable pool that contains vesicles available for exocytosis immediately after a Ca^2+^ increase (e.g., following an action potential), an asynchronous pool released more slowly after a Ca^2+^ increase, and a reserve pool that can replenish the releasable pools. Each of these pools is regulated by different subsets of proteins (Kaeser & Regehr 2014). Each ribbon synapse in a rod, cone, or BPC contains thousands of vesicles, and 80–95% of these vesicles are mobile in the cytoplasm (Holt et al. 2004, Rea et al. 2004). This contrasts with conventional synapses, which typically have only a few hundred or fewer vesicles. Furthermore, most of this cytoplasmic reserve pool at conventional synapses is immobilized by interactions with Synapsin 1 and 2 proteins, which link vesicles to the cytoskeleton in a Ca^2+^-dependent manner (Gitler et al. 2004).Since ribbon synapses lack synapsins (Mandell et al. 1990), it is reasonable to suggest that the unusually large fraction of mobile vesicles at retinal ribbon synapses is due to the absence of these molecules. However, transgenic expression of synapsin in photoreceptors resulted in proper targeting to synaptic vesicles but did not affect synaptic ultrastructure or light responses detected via electroretinograms (ERGs) (Geppert et al. 1994). Nonetheless, the large reservoir of mobile vesicles seems likely to be important for replenishment of ribbon-attached vesicle pools.

Release at rod, cone, and BPC synapses involves a subset of vesicles along the base of each ribbon. In rods and cones, the number of vesicles that make up the readily releasable pool that fuses immediately after Ca^2+^ influx matches the number of vesicles lining the bottom two rows of each ribbon (Bartoletti et al. 2010, Van Hook & Thoreson 2015). In BPCs, the fastest releasable pool in BPCs is roughly equivalent to only the bottom row (Mennerick & Matthews 1996, Singer & Diamond 2006). The remaining population of ribbon-attached vesicles in both photoreceptors and BPCs forms reserve pools that are released more slowly (Gomis et al. 1999, Heidelberger et al. 1994, Innocenti & Heidelberger 2008, Neves & Lagnado 1999, von Gersdorff & Matthews 1994). This general relationship between pools has been largely confirmed by direct imaging of synaptic vesicles (Lagnado et al. 1996, Llobet et al. 2003, Zenisek et al. 2000). Restoration of the ribbon-attached reserve pool presumably requires replenishment from cytoplasmic vesicles (Gomis et al. 1999, Holt et al. 2004).

The molecular underpinnings of how vesicles associate with ribbons remain poorly understood. Electron micrographs show that each vesicle is attached to a ribbon by 3–5 filaments (Usukura & Yamada 1980). Vesicles depart ribbons by fusion at the base and rarely, if ever, detach from the face (Jackman et al. 2009, Vaithianathan et al. 2016, Wen et al. 2018, Zenisek 2008). Individual tethers therefore need low-affinity interactions with vesicles to allow movement along the ribbon surface (Graydon et al. 2014) but exert sufficient combined force to prevent vesicles from detaching altogether. There could possibly be low-affinity interactions between the Ribeye B domain and lysophospholipids on vesicle membranes (Schwarz & Schmitz 2017). However, such low-affinity interactions would not be sufficient to impart specificity, since lysophospholipids are present in the membranes of many other organelles, including endosomes and Golgi-derived vesicles.

To ensure that synaptic vesicles, but not other organelles, remain attached, initial nonspecific interactions may need to be strengthened by specific protein–protein interactions. A possible candidate for higher-affinity interactions between vesicles and ribbons is the small GTP-binding protein RAB3A, which is present on the vesicle membrane. The GTP-bound form of RAB3A can interact with the large multidomain active zone proteins, RAB-interacting molecule (RIM) 1 and RIM2, and also associates with ribbons in a GTP-dependent manner. These findings prompted the hypothesis that vesicles might attach to ribbons via interactions between RAB3A on vesicles and RIM proteins on ribbons (Tian et al. 2012). Consistent with this hypothesis, introducing a RAB3A mutant protein that cannot hydrolyze GTP speeds the rundown of release in rods. However, RIM1 transcript levels are low in rods and cones (Figure 1), and early reports of RIM1 on photoreceptor ribbons appear to have been due to cross-reactivity of RIM1 antibodies with Piccolino (Lohner et al. 2017). RIM2 is concentrated at the base of the ribbon, inconsistent with a role in tethering vesicles along the ribbon face but perhaps suggesting a role in stabilizing vesicles near the membrane (tom Dieck et al. 2005).

Another possible facilitator of interactions between vesicles and ribbons is the WD-repeat family protein Rabconnectin 3 (DMXL2). Rabconnectin 3 is enriched in ribbon-bearing cells, localizes to ribbons, and interacts with RAB3A (Dittrich et al. 2023). Rabconnectin 3 also associates with vesicular H^+^-ATPases that promote acidification and glutamate loading of synaptic vesicles (Einhorn et al. 2012). Such interactions might help to ensure that ribbon-attached vesicles have the necessary machinery to load with glutamate prior to release.

The reduced form of the metabolic coenzyme nicotinamide dinucleotide (NADH) promotes lipid binding to Ribeye (Schwarz & Schmitz 2017). Electron transfer during mitochondrial activity shuttles NAD between its reduced (NADH) and oxidized (NAD^+^) states. Thus, metabolic changes in the NAD^+^–NADH ratio may regulate vesicle attachment and ribbon replenishment.

### 2.3. Vesicle Priming

Vesicles captured from the cytoplasm must go through a molecular priming process to become release ready. Docking and priming of vesicles requires the formation of SNARE (soluble NSF attachment protein receptor) complexes from interactions between the vesicle-associated SNARE (v-SNARE) synaptobrevin (also known asVAMP) and the target membrane SNAREs (t-SNAREs) STX and SNAP-25 on the plasma membrane (Rizo 2022). At most synapses, the STX-binding protein MUNC13 is essential for priming because it pulls the Habc N-terminal domain of STX away from the H3 domain to form an open configuration. This exposes an α-helical domain of STX, allowing interactions with α-helical SNARE domains of SNAP-25 and synaptobrevin and the formation of the SNARE complex.

MUNC18 is structurally unrelated to MUNC13 but shares a similar name because both were initially identified in *Caenorhabditis elegans* screens for uncoordinated mutants. MUNC18 is strongly expressed at both ribbon and conventional synapses, where it helps assemble the SNARE complex and stabilize the open configuration of STX. Formation of the SNARE complex brings the vesicle and plasma membranes closer to one another, readying the vesicle for fusion. Merger of the plasma and vesicle membranes is promoted by zippering together of the SNARE proteins. It is hypothesized that, in the absence of Ca^2+^, the Ca^2+^-binding protein synaptotagmin (SYT) restrains the SNARE complex from spontaneous fusion (Rizo 2022).

Vesicle priming at ribbon synapses differs from priming at conventional synapses and likely involves multiple steps. First and foremost, vesicle priming begins during residence on the ribbon. This was shown by selectively damaging ribbons using a technique known as fluorescence-assisted laser inactivation (FALI) (Snellman et al. 2011). In these experiments, the fluorophore fluorescein isothiocyanate (FITC) was attached to a peptide containing a consensus sequence that selectively binds CTBP proteins and thus attaches to Ribeye in ribbon-containing synapses. Photoactivation of FITC generates singlet oxygen molecules that cause highly localized damage. In both photoreceptors and BPCs, selectively damaging ribbons via FALI using a Ribeye-binding peptide had little effect on release evoked by the first stimulus following photodamage but substantially reduced subsequent release. The unaffected pool (i.e., the pool released by the first stimulus) was roughly equal to the number of vesicles on the ribbon. However, electron micrographs showed that, while subsequent release was impaired, the distribution of vesicles on the ribbon following FALI was unchanged. These results imply that previously primed vesicles in the readily releasable pool could be released, and new vesicles could reoccupy empty release sites, but these newly replenished vesicles were less capable of release, i.e., incompletely primed for release (Snellman et al. 2011). Introducing peptides into rod BPCs to inhibit the SNARE protein STX3B or SNAP-25 produced similar effects on release as damaging ribbons by FALI: Ribbon-associated vesicle pools could be released by the first stimulus, but subsequent release was impaired (Datta et al. 2017). This indicates the existence of a pool of vesicles resistant to new SNARE complex assembly that is similar in size to the one resistant to FALI. Given the hypothesized role of partial SNARE assembly arrested by SYT1 and complexin in vesicle priming, it is tempting to speculate that these two manipulations uncovered the same pool of preprimed vesicles.

A prerequisite for priming vesicles is disassembly of pre-existing SNARE complexes. This process requires ATP, and once again, vesicles in the ribbon-associated pool could be released from BPCs by the first stimulus applied after introducing a nonhydrolysable analog of ATP (ATPγS), but subsequent release was impaired (Heidelberger 1998). Eliminating ribbons altogether also impaired vesicle replenishment (Mesnard et al. 2022a). Taken together, these results provide strong support for the idea that vesicles are primed for release on the ribbon prior to reaching the active zone along the ribbon base.

Vesicle priming at ribbon synapses also involves atypical proteins. Three of four MUNC13 isoforms are expressed in neurons or neuroendocrine cells (MUNC13–1, 2, and 3). MUNC13–2 has two splice variants: brain specific (bMUNC13–2) and a more ubiquitously expressed variant (ubMUNC13–2). Most conventional synapses use MUNC13–1 or brain-specific MUNC13–2, but bipolar and photoreceptor ribbon synapses express ubMUNC13–2 (Cooper et al. 2012, Gierke et al. 2020, Schmitz et al. 2001). [Type 6 ON BPCs are an exception, expressing both MUNC13–2 and MUNC13–3 (Gierke et al. 2020).] Eliminating MUNC13 from conventional synapses in the brain that use STX1 eliminates docked vesicles and abolishes release, consistent with MUNC13 playing an essential role in vesicle priming at these synapses (Rizo 2022). However, genetic elimination of the MUNC13 protein from photoreceptor ribbon synapses (ubMUNC13–2) reduced the ERG b-wave by approximately 30% with no change in the number of membrane-associated vesicles in rods (Cooper et al. 2012). The b-wave of the ERG provides a measure of ON-type BPC responses, and thus, these modest effects contrast with the complete abolition of release at conventional synapses, suggesting that ubMUNC13–2 is not required for synaptic transmission from photoreceptors.

The putative priming molecules ubMUNC13–2 and RIM2 are both localized to the ribbon base. However, while there appears to be little, if any, RIM1 at photoreceptor ribbons (tom Dieck et al. 2005, Wang et al. 1997), elimination of RIM2 caused only small reductions in cone-driven light responses of HCs and scotopic b-wave amplitude (Lohner et al. 2017). Recruitment of MUNC13 to the active zone typically requires interactions with RIM proteins, but the RIM2 variant at photoreceptor ribbon synapses lacks an interaction domain for MUNC13 (Lohner et al. 2017). Together, these results suggest that interactions between RIM2 and ubMUNC13–2 are not essential for vesicle release from photoreceptors (Lohner et al. 2017). However, we note that eliminating both RIM1 and RIM2 from rods caused greater inhibition of rod Ca^2+^ currents and glutamate release than elimination of either alone (Grabner et al. 2015), raising the possibility that there may be sufficient RIM1 in photoreceptors to compensate for the loss of RIM2.

Like other synapses, retinal ribbon synapses use the SNARE proteins SNAP-25 and Synaptobrevin 2, but unlike conventional synapses, which typically use STX1, ribbon synapses in rods, cones, and BPCs use a retina-specific isoform, STX3B (Curtis et al. 2008, Morgans et al. 1996). The properties of STX3B suggest that there is an alternate priming mechanism at retinal ribbon synapses. Phosphorylation of residue T14 on the N terminus of STX3B by CaMKII promotes an open configuration, enhancing binding to SNAP-25 and thereby promoting SNARE complex formation (Gething et al. 2022, Liu et al. 2014). Phosphorylation of T14 also promotes fusion independent of its effect in promoting the open configuration of STX3B (Nishad et al. 2023). It has thus been hypothesized that STX3B may play a role like that of MUNC13 in vesicle priming (Liu et al. 2014). The t-SNAREs STX3B and SNAP-25 can both be found in vesicle membrane preparations isolated from retinal ribbon synapses, suggesting that both are present on ribbon-associated vesicles, along with the v-SNARE synaptobrevin (Liu et al. 2014). Consistent with the idea that Ca^2+^–CaM–CaMKII phosphorylation of STX3B might regulate priming, Ca^2+^ acting through calmodulin promotes vesicle replenishment at ribbon-associated sites (Gomis et al. 1999, Van Hook et al. 2014, von Gersdorff & Matthews 1997). In this scenario, vesicles descending the ribbon to approach Ca^2+^ channels at the base would be exposed to higher levels of Ca^2+^ that could facilitate CaMKII phosphorylation, opening of STX3B, and vesicle priming. Consistent with this, STX3B phosphorylation is enhanced by darkness in rods (when rods are maximally depolarized) and by light in rod BPCs (when these cells are maximally depolarized) (Campbell et al. 2020). The unphosphorylated form of STX3B can also form SNARE complexes, albeit with lower probability, and this could explain the presence of both Ca^2+^-dependent and Ca^2+^-independent components of vesicle replenishment (Babai et al. 2010, Gomis et al. 1999, Van Hook et al. 2014).

Ca^2+^-dependent priming has the benefit of allowing activity-dependent regulation of replenishment rates, adjusting priming rates to accommodate illumination-dependent changes in release. Priming vesicles during their descent down the ribbon also has the benefit of promoting the rapid fusion of newly arrived vesicles. Vesicles can fuse very rapidly (<60 ms) upon reaching the ribbon base in rods, consistent with the idea that most priming steps have already occurred by the time they reach the base (Chen et al. 2013). We note, however, that vesicles arriving beneath BPC ribbons pause for a longer time before fusion (approximately 250 ms) (Joselevitch & Zenisek 2020), and that this more closely resembles the delay seen with total internal reflection fluorescence (TIRF) imaging at conventional synapses (>200 ms) (Miki et al. 2020).

Adjacent vesicles that possess both v-SNAREs and t-SNAREs could potentially fuse with one another prior to fusing with the plasma membrane. This could provide a mechanism for synchronizing multivesicular release, as seen at rod, cone, and BPC ribbon synapses (Hays et al. 2020, Matthews & Sterling 2008, Singer et al. 2004). Consistent with this possibility is evidence that larger miniature excitatory postsynaptic currents that are thought to arise from synchronous multivesicular fusion in rods and BPCs are inhibited both by damaging ribbons via FALI and by introducing STX3 inhibitory peptides (Hays et al. 2020, Mehta et al. 2013).

### 2.4. Complexins: Key Regulators of Exocytosis

An important accessory member of the SNARE complex is complexin. Complexins are cytosolic proteins that bind to a groove in the SNARE complex and regulate fusion readiness. Complexins earned their name from specific interactions with formed SNARE complexes, but the name is also fitting given their complicated effects on synaptic vesicle exocytosis, with both a facilitatory N-terminal domain and an inhibitory accessory helix (AH) domain in mammalian isoforms (Trimbuch & Rosenmund 2016). In the absence of Ca^2+^, the AH domain is positioned near the vesicle membrane, inhibiting SNARE complex zippering and spontaneous fusion of vesicles. In the presence of Ca^2+^, this inhibitory effect is relieved, allowing the facilitatory effects of the N terminus to dominate.

Retinal ribbon synapses possess Complexin 3 and 4 isoforms (Reim et al. 2005). Complexin 3 is less common than Complexins 1 and 2 in the brain, and Complexin 4 is expressed selectively in the retina (Reim et al. 2005) (Figure 1). Complexin 3 is expressed in both photoreceptors and BPCs but less robustly in rods than in cones (Landgraf et al. 2012; Reim et al. 2005, 2009). Complexin 4 is expressed in photoreceptor cells and a handful of cone BPCs (Reim et al. 2005, 2009).

Inhibiting Complexin 3 with inhibitory peptides or eliminating it altogether increased spontaneous release and reduced evoked release in retinal BPCs (Mortensen et al. 2016; Vaithianathan et al. 2013, 2015). Eliminating both Complexins 3 and 4 halved b-waves (which reflect ON BPC responses) in mouse retina with little effect on a-waves (which measure photoreceptor light responses), suggesting that the complexins play a role in regulating release from photoreceptors (Reim et al. 2009). Consistent with this, loss of Complexins 3 and 4 also elevated levels of spontaneous release and inhibited evoked release in cones (Babai et al. 2016). In vitro fusion assays confirmed the ability of Complexins 3 and 4 to inhibit fusion involving the retina-specific SNARE STX3B (Nishad et al. 2023). These results are not dissimilar to the behavior of Complexins 1 and 2 in other neurons, which shows facilitation of evoked release by the N-terminal domain and inhibition of spontaneous release by the AH domain (Trimbuch & Rosenmund 2016).

Unlike Complexins 1 and 2 of vertebrates, but like invertebrate complexins, Complexins 3 and 4 possess C-terminal CAAX-box consensus sequences for a post-translational lipid modification that promotes membrane interactions and synaptic localization (Reim et al. 2005). This CAAX-box helps localize complexin to membranes and thus facilitates efficient interactions between it and SNARE complexes of recently arrived vesicles. This may in turn help ensure that vesicles primed during descent down the ribbon do not fuse prematurely.

### 2.5. Synaptotagmins: Ca^2+^ Sensors That Mediate Evoked Release

The final step in SNARE-mediated fusion is triggered by Ca^2+^ binding to SYT. There are 17 SYT homologs in mammals, but only 8 bind Ca^2+^: SYT1–3, 5–7, 9, and 10. SYT1, 2, and 9 show the lowest Ca^2+^ affinities (EC50 approximately 10–20 μM), whereas SYT3 and 7 show the highest (Sugita et al. 2002).

Different sensors can regulate different phases of synaptic vesicle release. In most central nervous system neurons, fast release that is tightly synchronized to nearby Ca^2+^ channel openings is controlled primarily by SYT1, 2, and 9 (Xu et al. 2007). A higher-affinity sensor, SYT7, regulates the slower, asynchronous form of release that is less tightly coupled to individual channel openings (MacDougall et al. 2018). Control of this type of asynchronous release has also been ascribed to a cytosolic protein, Doc2 (Yao et al. 2011).

The prototypical SYT1 has two Ca^2+^-binding domains in which three Ca^2+^ ions bind the C2A domain of SYT1, and two Ca^2+^ ions bind the C2B domain. Ca^2+^ binding to these domains neutralizes negative aspartate residues on SYT1, allowing interactions with negatively charged phospholipid head groups on the membrane (Rizo 2022). These interactions promote zippering of the SNARE complex and release of inhibitory interactions with complexins, lowering the energy barrier for fusion.

SYT1 is expressed at the ribbon synapses of rods, cones, and many BPCs in mammalian retina. A subset of cone BPCs express SYT2 (Berntson & Morgans 2003, Fox & Sanes 2007, Heidelberger et al. 2003). Conditional knockouts of SYT1 show that this sensor regulates fast synchronous release of the readily releasable vesicle pool in rods and cones (Grassmeyer et al. 2019, Mesnard et al. 2022b). Loss of SYT1 from rods also elevated rates of spontaneous release consistent with a proposed clamping function for SYT1. However, loss of SYT1 from cones did not change spontaneous rates, suggesting that unclamping the SNARE apparatus may not be the principal mechanism by which SYT1 promotes vesicle fusion, at least in these cells (Rizo 2022).

Consistent with a possible role for SYT7 in regulating asynchronous release (MacDougall et al. 2018), eliminating it from rod BPCs or rods abolished release from nonribbon sites that could be evoked only by lengthy depolarization (Luo et al. 2015, Mesnard et al. 2022b). However, despite these effects on slow release, eliminating SYT7 had no effect on ERG b-waves. Instead, b-waves were abolished solely by eliminating SYT1 from rods and cones (Mesnard et al. 2022b). Together with evidence that SYT7 is expressed at low levels in photoreceptors (Mesnard et al. 2022b) (Figure 1), this result suggests a subtle modulatory role for SYT7. SYT7 shows higher Ca^2+^ affinity than SYT1, and thus, the spread of Ca^2+^ to distant release sites during a strong, sustained depolarizing stimulus may favor SYT7-mediated release, while normal physiological fluctuations in Ca^2+^ may not reach these distant sites. SYT7 participates with SYT1 in clamping SNARE complexes (MacDougall et al. 2018) and thus might also mediate more significant fusion when SYT1 is absent.

Unlike mammalian rods and cones, the photoreceptors of fish and amphibians do not use SYT1 (Berntson & Morgans 2003, Heidelberger et al. 2003). Experiments on salamander rods and cones using caged Ca^2+^ compounds showed that exocytosis could be evoked by submicromolar Ca^2+^ with a cooperativity of *n* = 2–3 (Duncan et al. 2010, Thoreson et al. 2004). By contrast, conventional synapses using SYT1 typically show a much lower Ca^2+^ affinity and steeper Ca^2+^ dependence, with cooperativity of *n* = 4–5 (i.e., release increases with the fourth or fifth power of [Ca^2+^]_i_) (Dodge & Rahamimoff 1967, Heidelberger et al. 1994, Schneggenburger & Neher 2000). This cooperativity is thought to arise from sequential binding of a total of five Ca^2+^ ions, three to the C2A domain and two to the C2B domain. In the zebrafish retina, it has been proposed that the retina-specific *syt5* might replace or work with SYT1 as a Ca^2+^ sensor for fast synchronous release (Henry et al. 2022). The weak Ca^2+^ cooperativity for release from amphibian photoreceptors may involve different SYT isoforms, but lipid composition and accessory proteins can also modify Ca^2+^-binding properties of SYT. For example, membranes with acidic phospholipids, in particular, PI(4,5)P2, can increase Ca^2+^ affinity (Bai et al. 2004), and phosphomimetic mutants of SNAP-25 alter Ca^2+^ sensitivity (Sorensen et al. 2002).

The double C2 domain cytosolic protein DOC2 has been proposed to be a sensor for both spontaneous and asynchronous release (Groffen et al. 2010, Yao et al. 2011; but see Diez-Arazola et al. 2020). Both isoforms, DOC2a and DOC2b, bind Ca^2+^ at the tandem C2 domain. This binding is thought to promote a superprimed state of the SNARE complex (Xue et al. 2018) that facilitates spontaneous fusion. It has been suggested that DOC2a selectively regulates spontaneous release at excitatory synapses, whereas DOC2b regulates spontaneous release at inhibitory synapses (Courtney et al. 2018). However, DOC2b transcript levels are considerably higher than those of DOC2a at glutamatergic rod and cone synapses, while levels of DOC2a are higher at GABAergic synapses of HCs.

### 2.6. Voltage-Gated Ca^2+^ Channels

The properties of Ca^2+^ channels in the retina have been reviewed elsewhere (Pangrsic et al. 2018, Williams et al. 2022), so we only summarize some key features. The principal channels that regulate synaptic transmission at the ribbon synapses of rods and cones are L-type Ca^2+^ channels, rather than the N- or P-type channels used at most synapses. The Ca_V_1.4 L-type channels in rods and cones are selectively expressed in the retina. BPCs have both Ca_V_1.3 and Ca_V_1.4 channels, along with Ca_V_2 and Ca_V_3 channels, as discussed further below (Zhang et al. 2022). Ca_V_1.3 and Ca_V_1.4 L-type channels show less inactivation than other Ca^2+^ channels, helping them remain active at these continuously active synapses. This lack of inactivation is particularly prominent in splice variants of Ca_V_1.3 and Ca_V_1.4 L-type channels, which have a long C terminus with an autoinhibitory domain. This autoinhibitory domain competes with apo-CaM (Ca^2+^-free CaM) for attachment to a proximal C-terminal region that normally mediates Ca^2+^-dependent inactivation. Short C-terminal splice variants that lack this autoinhibitory region show greater Ca^2+^-dependent inactivation (Haeseleer et al. 2016, Singh et al. 2008, Tan et al. 2011). Splice variants can also vary in voltage dependence (Haeseleer et al. 2016, Hofer et al. 2021, Lieb et al. 2012, Singh et al. 2008, Williams et al. 2018).

The Ca^2+^-binding protein CABP4 is also selectively expressed in rods and cones, where it plays an important role in regulating Ca_V_1.4 voltage dependence (Haeseleer et al. 2004, Shaltiel et al. 2012). Most L-type channels are barely active over the physiological voltage range of rods and cones, between −35 and −65 mV. Interactions between CABP4 and the C-terminal domain of Ca_V_1.4 cause a negative activation shift that improves the overlap between activation and physiological voltage range (Haeseleer et al. 2004, 2016; Shaltiel et al. 2012). Truncating the C terminus of Ca_V_1.4 to remove the autoinhibitory domain also shifts activation approximately 5 mV in the negative direction, suggesting that this region mediates the CABP4-induced shift.

Intracellular accessory β subunits help traffic α1 subunits from the endoplasmic reticulum but also modify voltage dependence. The principal β subunit in photoreceptors is β2 (Ball et al. 2002, Katiyar et al. 2015). β2 Subunits are also the most common in BPCs, with β4 being the second most common (Zhang et al. 2022).

α2δ subunits associate with the extracellular surface of Ca^2+^ channels via a GPI link and are important for delivering Ca^2+^ channels to the synapse. α2δ4 is the major accessory α2δ subtype in photoreceptors (Kerov et al. 2018, Wang et al. 2017, Wycisk et al. 2006) and is also present in BPCs (Zhang et al. 2022). Different types of BPCs can also express various combinations of α2δ1, α2δ2, and α2δ3 (Zhang et al. 2022). α2δ4 participates in trans-synaptic interactions with ELFN1 in rod BPCs that are essential for establishing proper synaptic connections between these cells (Wang et al. 2017).

In addition to L-type channels, bipolar cells also express N- and P-type Ca_V_2 channels in various combinations among different cell types (Zhang et al. 2022). Ca_V_2 channels show more pronounced inactivation than Ca_V_1.3 and Ca_V_1.4 channels, making their responses more transient. Accordingly, transcript levels for Ca_V_2 are generally higher in the middle layers of the inner plexiform layer, where terminals of BPCs with more transient responses are located. The expression of different Ca^2+^ channel subunits may sculpt differences in response kinetics among BPC subtypes that are initiated in the outer plexiform layer (DeVries 2000, Grabner et al. 2023).

Ca_V_3 T-type channels show even greater transient activation kinetics than Ca_V_2 channels. T-type channels can be found in BPCs (Pan et al. 2001, Singer & Diamond 2003, Zhang et al. 2022), and there is recent evidence for Ca_V_3.2 channels in mouse cones (Davison et al. 2022). Ca_V_3.2 channel transcript levels are generally higher in transient bipolar types than in other bipolar cell types (Zhang et al. 2022). Ca_V_3 channels activate at more negative membrane potentials than Ca_V_1 and Ca_V_2 channels and thus should normally be inactive at cone and BPC resting potentials (−40 to −60 mV).However, strong hyperpolarization can relieve inactivation, allowing T-type currents to boost transient depolarizing responses of OFF BPCs or cones at light offset and ON BPCs at light onset.

### 2.7. Other SNARE-Related Proteins

Several other SNARE-related proteins are expressed at high levels in photoreceptors and/or BPCs. For example, STXBP5 (tomosyn) shows higher transcript levels in rods, cones, and cone BPCs compared to the brain (Figure 1). Tomosyn is thought to serve as a decoy SNARE by interacting with STX1 proteins that are not complexed with UNC13 or UNC18 (Sauvola & Littleton 2021), thereby limiting UNC13/18-independent priming.Tomosyn can also interact with STX3B (Madera-Salcedo et al. 2018) and thus might play a similar role at retinal ribbon synapses.

The synaptic vesicle protein SV2 has three isoforms; SV2B is the major isoform at retinal ribbon synapses, but cones and a subset of BPCs also possess SV2A. SV2 is a member of the transporter superfamily, although there is no clear evidence supporting its transport function. It has instead been suggested that the large intravesicular loops of SV2 may provide structural support to other transmembrane vesicle proteins (Stout et al. 2019). Loss of SV2B from rod BPCs increases presynaptic Ca^2+^ levels (Wan et al. 2010), and eliminating SV2B from the retina causes a modest reduction in ERG b-waves, suggesting reduced release from photoreceptors. While this might be due to changes in intracellular Ca^2+^, loss of SV2B also reduces SYT1 expression (Lazzell et al. 2004).

UNC119 is strongly expressed in photoreceptors (Higashide et al. 1998, Swanson et al. 1998), where it helps transport lipidated proteins through the cilium connecting the inner and outer segments (Frederick et al. 2020). UNC119 is also present at photoreceptor ribbon synapses (Higashide et al. 1998), where it interacts with Ribeye and CABP4 (and perhaps Complexin 4) through a prenyl-binding protein homology domain (Alpadi et al. 2008, Haeseleer 2008). Eliminating UNC119 causes slow retinal degeneration (Ishiba et al. 2007), but prior to degeneration, rod BPCs show diminished light sensitivity with no change in photoreceptor sensitivity (Fehlhaber et al. 2023). It was therefore suggested that UNC119 might enhance the transmission of rod signals (Fehlhaber et al. 2023). However, UNC119 can inhibit GTPases, including dynamin, and inhibition of endocytosis (Karim et al. 2010) can also diminish release (Wen et al. 2018).

Several multidomain proteins are important to ensuring the proper location of synaptic proteins (for a review, see Moser et al. 2020). Rod and cone ribbons are anchored to the membrane by a trough-like structure at their base, known as the arciform density. The arciform density contains Bassoon, RIM2, MUNC13–2, and Ca_V_1.4 proteins aligned in two parallel rows (Grabner et al. 2022). Expression of a truncated form of Bassoon causes ribbons to detach from the membrane (Dick et al. 2003, Ryl et al. 2021). ELKS (ERC1) and CAST (ERC2) are key cytomatrix constituents of the active zone at most synapses (Rizo 2022). CAST also associates with the arciform density, and loss of this protein disrupts ribbons, inhibits Ca^2+^ currents, and reduces ERG b-waves (Hagiwara et al. 2018, tom Dieck et al. 2012). Loss of ELKS causes fewer deficits than loss of CAST (Hagiwara et al. 2018).

Curiously, the postsynaptic density protein-95 (PSD-95) is prominently expressed presynaptically in rod and cone terminals. In cones, PSD-95 associates with scaffolding proteins that are also normally located postsynaptically, including Shank1A (Stella et al. 2012), MPP4, DLG1, and VELI3 (Aartsen et al. 2006). This protein complex helps to ensure synaptic localization of the plasma membrane Ca^2+^-ATPase (PMCA) (Aartsen et al. 2009, Yang et al. 2007). The PMCA is the principal mechanism for extrusion of synaptic Ca^2+^ in rods (Aartsen et al. 2009, Johnson et al. 2007, Yang et al. 2007).

### 2.8. Endocytosis at Retinal Ribbon Synapses

Ongoing synaptic vesicle release must be balanced by endocytosis to restore the pool of available vesicles and avoid distortions in the synaptic terminal membrane that can impair release (Wen et al. 2018). The problem of balancing release and retrieval is shared by many neurons, but the unceasing release of vesicles at ribbon synapses presents a particular challenge. Studies using a variety of techniques, including membrane capacitance measurements, optical imaging, and rapid freeze transmission electron microscopy, show that at least five mechanisms are available for retrieving synaptic vesicles (Gan & Watanabe 2018): (*a*) ultrafast retrieval (tau < 1 s), (*b*) fast compensatory endocytosis (tau = 1–2 s), (*c*) bulk retrieval, (*d*) slow clathrin-mediated endocytosis, and (*e*) kiss and run (<1–2 s).

In many of the first studies that described mechanisms for fast endocytosis, bulk retrieval and clathrin-mediated endocytosis were performed using ribbon synapses of goldfish BPCs (Holt et al. 2003, Jockusch et al. 2005, Llobet et al. 2011, Logiudice & Matthews 2007, Logiudice et al. 2009, von Gersdorff & Matthews 1994). All three modes appear to require the GTPase dynamin for scission of newly formed endocytic vesicles, although there is also evidence that internalization may proceed in the absence of GTP hydrolysis (Heidelberger et al. 2002). Fast endocytosis in BPCs uses a retina-specific splice variant of a BAR domain protein, Amphiphysin 1 (Hosoya et al. 2004, Jockusch et al. 2005), which, like other BAR domain proteins, has a curved structure that facilitates membrane bending. Slow clathrin-mediated endocytosis in BPCs requires clathrin and its adapter protein, AP2, but not amphiphysin (Jockusch et al. 2005, Logiudice et al. 2009).

Syndapin (PACSIN1) is another BAR domain protein that cooperates with dynamin to facilitate vesicle fission during endocytosis. By inhibiting fission, eliminating syndapin causes an increase in omega figures (i.e., hemifused vesicle intermediates) and an accumulation of endosomes at rod synapses (Koch et al. 2011).

The endocytic proteins dynamin, syndapin, and amphiphysin cluster together in the perisynaptic zone of rod ribbon synapses, suggesting that this is the main region of vesicle retrieval (Fuchs et al. 2014, Wahl et al. 2013). Membrane capacitance (C_m_) recordings from salamander rods and cones showed both slow and ultrafast forms of endocytosis (Innocenti & Heidelberger 2008, Van Hook & Thoreson 2012). Endocytosis was blocked by dynamin inhibitors in rods, but dynamin and GTPase inhibitors failed to block ultrafast endocytosis in cones (Van Hook & Thoreson 2012).

One mechanism for ultrafast endocytosis is kiss and run, in which vesicles form a transient fusion pore, allowing neurotransmitter release without proceeding to full-collapse fusion with the membrane. Kiss and run is important for regulating the release of small molecules from large, dense core vesicles (Alvarez de Toledo et al. 1993), but whether it contributes significantly to the release of small, clear vesicles in neurons remains controversial (Chanaday et al. 2019). The ability of small synaptic vesicles in neurons to participate in kiss and run is supported by high-resolution single-vesicle membrane capacitance measurements (He et al. 2006). Optical studies comparing the uptake of large and small dye molecules suggested that over half of release events at rod and cone ribbon synapses in the amphibian retina may result from kiss and run (Wen et al. 2017). However, differences in uptake and retrieval might also be due to dye properties other than the hydrodynamic radius.

Another ultrafast mode discovered using rapid-freeze electron microscopy begins with the rapid retrieval of 60–80-nm-diameter endosomes (Watanabe et al. 2013). These endosomes later merge into larger endosomes, and while clathrin is not involved in the initial retrieval step, it contributes to regenerating synaptic vesicles from these large endosomes (Gan & Watanabe 2018, Watanabe et al. 2014). Two populations of clathrin heavy chain molecules are found in mouse rods, one in the periactive zone and the other deeper in the cell, raising the possibility that clathrin might be present on the endosomal membrane (Wahl et al. 2013). TIRF microscopy experiments in zebrafish retinal BPCs using fluorescently tagged clathrin also showed the presence of clathrin on endosomes or vesicles near release sites, potentially consistent with this form of ultrafast retrieval (Pelassa et al. 2014).

Photoreceptors are also capable of slow forms of endocytosis (Innocenti & Heidelberger 2008, Wen et al. 2017). Electron micrographs show clathrin-coated vesicles along the perisynaptic membrane near photoreceptor ribbons, consistent with clathrin-mediated endocytosis (Fuchs et al. 2014, Gray & Pease 1971, Spiwoks-Becker et al. 2001). Bulk endocytosis may also contribute to vesicle retrieval by photoreceptors. Following 3 h of darkness (when release from rods is maximal), dense clusters of endosomes emerge in the perisynaptic region of rods (Fuchs et al. 2014). These clusters contain key endocytic proteins and are not seen in light-adapted retina. While bulk retrieval is generally thought to contribute to membrane retrieval under nonphysiological conditions of overstimulation (Gan & Watanabe 2018), these dense endosomal clusters may represent a novel form of bulk retrieval used under normal conditions.

Synaptojanin helps uncoat clathrin-coated synaptic vesicles and plays a role in ultrafast endocytic retrieval (Watanabe et al. 2018), as well as endolysosomal trafficking (Gad et al. 2000). Eliminating synaptojanin in zebrafish disrupted cone synapses and led to detached ribbons, although it had fewer effects on rods (George et al. 2014, Holzhausen et al. 2009, Van Epps et al. 2004).

### 2.9. Disease-Related Mutations in Synaptic Proteins

Mutations in synaptic proteins cause many retinal and neurological defects. Because of their essential roles in synaptic release throughout the body, homozygous mutations of SNARE proteins are often lethal. Disease-related mutations of SNARE proteins are thus typically heterozygous, with the mutant allele acting in a dominant negative fashion or by reducing activity of the normal allele (i.e., haploinsufficiency). As has been recently reviewed (Frederick & Zenisek 2023), mutations in various ribbon-associated proteins can lead to retinal dystrophies, including congenital stationary night blindness (e.g., Ca_V_1.4 mutations) and cone–rod dystrophies (e.g., UNC119, RIM1, CABP4). STX3 mutations cause retinal dystrophies by interfering with ciliary trafficking to the outer segment (Janecke et al. 2021, Kakakhel et al. 2020).

## 3. HORIZONTAL CELLS

HCs are GABAergic interneurons that mediate lateral inhibition among photoreceptors and feed-forward inhibition onto BPCs (Hirano et al. 2020, Thoreson & Mangel 2012). Unlike canonical neurons, HCs are nonspiking neurons and release neurotransmitters from their dendrites. Despite these unconventional features, they have a relatively conventional array of synaptic proteins (Figure 1).

Electron micrographs of HCs in various species typically show only a few small, clear vesicles, contrasting with the abundance of vesicles in neighboring photoreceptor terminals. Studies on HCs from nonmammalian retinas showed that GABA release involved Ca^2+^-independent, Na^+^-dependent actions of reversed GABA transport (Schwartz 2002). Together, these results suggested that release from HCs did not involve SNARE-dependent vesicle fusion. However, subsequent studies showed that, unlike in cold-blooded vertebrates, GABA release from mammalian HCs was Ca^2+^ dependent and regulated by L, N and P/Q Ca^2+^ channels (Hirano et al. 2020, Liu et al. 2013). Ca^2+^ influx through these channels acts on SYT2 to promote SNARE-dependent fusion (Fox & Sanes 2007, Lee & Brecha 2010). HCs possess a conventional array of SNARE proteins, including SNAP-25, STX1A, and Synaptobrevin 1 (VAMP1), along with SV2A and Complexins 1 and 2 (Guo et al. 2009, 2010; Hirano et al. 2005, 2007, 2011). Like other conventional synapses, HCs also express Synapsin 1 (Hirano et al. 2005, Lee & Brecha 2010), suggesting that vesicle recruitment makes use of similar mechanisms to those used by conventional action-potential firing neurons of the brain and neuromuscular junction.

The presence of conventional SNAREs suggests that feedforward GABAergic inhibition from HCs to BPC dendrites operates in a conventional manner. By contrast, feedback inhibition to rods and cones involves unconventional mechanisms (Thoreson & Mangel 2012). While extracellular changes in voltage produced by ephaptic currents have been proposed to contribute to inhibitory feedback from HCs to cones, the essential mechanism involves pH modulation of presynaptic Ca^2+^ channels on rod and cone terminals (Hirasawa & Kaneko 2003, Warren et al. 2016b). How can light-evoked changes in HC membrane voltage regulate cleft pH? Na^+^/H^+^ exchangers extrude protons generated by metabolic activity into the synaptic cleft between HCs and photoreceptors (Warren et al. 2016a). When HCs hyperpolarize to light, cleft pH rises as protons are buffered by an attendant rise in cleft bicarbonate (Grove et al. 2019, Wang et al. 2014, Warren et al. 2016a). This rise in bicarbonate appears to involve movement through GABA receptor anion channels, driven by the outward anion driving force produced by light-evoked hyperpolarization (Grove et al. 2019). In this scenario,GABA does not regulate postsynaptic conductances in photoreceptors directly, as is expected for a conventional inhibitory neurotransmitter. Instead, GABA maintains a population of open anion channels that allows transmembrane bicarbonate flux to regulate cleft pH and thus the activity of presynaptic Ca^2+^ channels. Given the small volume of the synaptic cleft, bicarbonate efflux through a few unblocked anion channels may be enough to support feedback.

## 4. AMACRINE CELLS

ACs constitute a diverse set of axonless interneurons that modulate signaling between bipolar and ganglion cells. They receive synaptic input from BPCs, and most provide glycinergic or GABAergic inhibition to BPCs (mediating local feedback and lateral inhibition), retinal ganglion cells (mediating feedforward inhibition), or other ACs (providing serial inhibition). In the mouse retina, 63 different types of ACs have been identified based on their molecular composition (Yan et al. 2020). Each AC has a unique role in shaping signals that is critical to signaling specific aspects of vision by different ganglion cell types. While most release GABA or glycine, many have also been shown to release acetylcholine, dopamine, glutamate, and various neuropeptides (Diamond 2017). Amacrine presynaptic sites are morphologically conventional, with a collection of vesicles near active zones and without a ribbon, but unconventional in that many are nonspiking, release occurs at dendritic locations, signaling can be tonic, and the sources of Ca^2+^ for driving release seem to be more diverse than in other cell types.

Owing in large part to the diversity of this class of neuron, comparatively less is known about the features of neurotransmitter release from these cells compared to other retinal cells. However, single-cell RNA sequencing (RNAseq) experiments have yielded detailed expression profiles of the many cell types, and general properties can be gleaned from the data. Based on these data, ACs have a conventional array of presynaptic proteins. For example, despite being dendritic rather than axonal in nature, ACs make use of canonical neuronal SNARE proteins with high expression of STX1 and STX2 (Sherry et al. 2006), VAMP2, and SNAP-25 along with Complexins 1, 2, and 3 (Lux et al. 2021, Reim et al. 2005). In addition, unlike ribbon synapses, seemingly all ACs express Synapsin 1 and/or Synapsin 2. ACs also express SYT1, along with the active zone proteins Piccolo and Bassoon (Yan et al. 2020). While much remains to be learned about the synaptic properties of individual cell types, a few representative subtypes of ACs have been studied in detail at the synaptic level.

### 4.1. AII Amacrine Cells

AII ACs play a critical role in relaying visual information from rod BPCs to ganglion cells via an unconventional circuit. AII ACs use gap junctions to drive changes in neurotransmitter release from cone BPCs, while also driving glycine release from lobular appendages in the OFF layer that provide parallel signals to the OFF pathway (Demb & Singer 2012). As in ribbon-type synapses, glycine release from lobular appendages is triggered by Ca^2+^ entry through slowly inactivating Ca_V_1.3 L-type Ca^2+^ channels (Habermann et al. 2003).

Capacitance recordings from AIIs revealed some unexpected properties of exocytosis. First, reducing extracellular Ca^2+^ reduced neurotransmitter release in a nearly linear fashion, similar to results in photoreceptors but different from most other neurons. Moreover, despite having conventional synapses lacking ribbons, the kinetics of exocytosis in AIIs are similar to those in ribbon synapses, and AII amacrine cells show the ability to release continuously when challenged with lengthy depolarization (Balakrishnan et al. 2015). These properties fit the role that these cells play in tonic inhibitory signaling in the retina and provide further evidence that ribbons are not required for tonic release, consistent with findings from Ribeye knockout mice.

The molecules that give rise to these photoreceptor-like properties of release remain unknown. Single-cell RNAseq data show that AII ACs express the conventional SNAREs STX1b, SNAP-25, and VAMP2, as well as SYT1 and CPLX1, suggesting that they operate in a conventional fashion similar to neurons in other parts of the brain. However, AIIs also express STX3 and are among the few ACs that abundantly express CPLX3, suggesting that certain aspects of vesicle priming and delivery are tailored to the particular needs of this cell (Yan et al. 2020). For example, lipid binding properties of the CPLX3 C terminus may facilitate fast and efficient recruitment to synapses by restricting movement of these molecules to the plane of the membrane, rather than allowing them to move freely throughout the entire terminal.

### 4.2. A17 Amacrine Cells

A17 ACs are also an important part of the visual circuit, responsible for transmitting signals in low light. Rod BPCs release glutamate onto these cells, which then provide local, reciprocal GABAergic feedback to the same BPC.

The release of GABA requires Ca^2+^ but makes use of a combination of Ca^2+^ entry via NMDA receptors, voltage-gated Ca^2+^ channels, and Ca^2+^ stores activated by Ca^2+^-induced Ca^2+^ release (Chavez & Diamond 2008, Chavez et al. 2006). Pharmacological blockade of different Ca^2+^ sources indicates that release is most sensitive to blockers of NMDA receptors, N-type Ca^2+^ channels, and internal Ca^2+^ stores. Interestingly, blockers of L-type, T-type, and P/Q-type Ca^2+^ channels also reduced synaptic responses, albeit more modestly, indicating that GABA release from A17s is driven by a complex interplay among multiple Ca^2+^ sources.

Reciprocal feedback from A17 cells to tonic ribbon-type rod BPCs is expected to provide tonic release over prolonged periods, as is the case in ribbon synapses. Indeed, recordings from rod BPCs reveal prolonged inhibitory postsynaptic responses in response to activation of A17s. As with other AC types, A17s express abundant neuronal SNAREs, as well as CPLX1 and SYT1. They also express higher levels of SYT7 than most other retinal neurons. Along with AIIs, A17s are among those that express the highest levels of CPLX3 (Yan et al. 2020).

### 4.3. Starburst Amacrine Cells

SACs have been intensely studied for their roles in signaling direction selectivity (Wei 2018) and propagation of retinal waves during critical periods of retinal development (Feller et al. 1996, Zhou 1998). They are unique in that they are the only cholinergic ACs found in the retina. They release both GABA and acetylcholine from their dendrites onto postsynaptic dendrites of direction-selective ganglion cells. Differences in the placement and timing of acetylcholine and GABA release are important for shaping the direction-selective responses of these cells (Wei 2018).

SACs release GABA and acetylcholine with different sensitivities to Ca^2+^ and Ca^2+^-channel blockers, indicating that the GABA and acetylcholine are released from different vesicles (Lee et al. 2010). A specific N-type channel blocker eliminated cholinergic transmission while reducing GABAergic transmission by one-third, whereas a P/Q blocker reduced GABAergic transmission by half, with a lesser effect on cholinergic transmission. Moreover, cholinergic transmission exhibits facilitation with repetitive stimuli, while GABAergic transmission does not (Lee et al. 2010). The ability of these cells to regulate cholinergic and GABAergic transmission independently improves their ability to sculpt responses of direction-selective ganglion cells to changing conditions of illumination. As with other ACs, the molecules that drive release are largely conventional. ACs show strong expression of neuronal SNAREs, as well as Synapsin 1, Synapsin 2, SYT1, and Complexin 2. SACs have high levels of amphiphysin (STXBP6) that, like tomosyn (STXBP5) at ribbon synapses, may compete with synaptobrevin and serve as a decoy SNARE to limit promiscuous priming of unproductive SNARE complexes (Kondratiuk et al. 2020).

One unusual feature of SACs among ACs is that they are the only ACs to express RIM1 at high levels (Yan et al. 2020). The significance of this is unclear. At the calyx of Held, which expresses both RIM1 and RIM2, these proteins appear to be largely redundant, although elimination of RIM2 had a slightly larger effect on the presynaptic calcium current (Han et al. 2015). However, both proteins exhibit multiple splice forms that may allow differential regulation of synaptic release in different cell types.

### 4.4. vGlut3 Amacrine Cells

vGlut3 ACs are the only glutamatergic ACs in the retina, providing excitatory glutamatergic input to many different ganglion cells, as well as some BPCs and ACs (Kim et al. 2015, Lee et al. 2014). vGlut3 ACs also provide inhibitory glycinergic input to many cells, including ON direction-selective ganglion cells involved in optokinetic nystagmus (Mani et al. 2023) and suppressed-by-contrast M1 intrinsically photosensitive ganglion cells (Lee et al. 2021, Tien et al. 2016).

The sources of Ca^2+^ that drive the release of these different transmitters remain unknown, but vGlut3 cells express both L-type and T-type Ca^2+^ channels, as well as NMDA- and AMPA/kainate-type glutamate receptors (Grimes et al. 2011). The array of presynaptic proteins in vGlut3 ACs are largely consistent with those at conventional synapses, with strong expression of neuronal SNAREs, Complexin 2, and SYT1. Whether differential expression of synaptic proteins within vGlut3 ACs is important for differential regulation of glycine and glutamate release remains unclear.

## 5. SUMMARY

The retina detects and encodes visual information for higher-order processing in the brain. To fulfill this task, the retina uses diverse circuits to process different features of the visual world originally encoded as graded changes in photoreceptor membrane potential and subsequently converted into a series of spikes by specialized types of ganglion cells. To do so, the retina has evolved synapses with molecular and anatomical specializations whose functions are incompletely understood. Most neurons of the retina upstream of ganglion cells either are nonspiking or exhibit spikes that fail to propagate across the entire cell. Photoreceptors and most BPCs make use of graded changes in membrane potential to regulate voltage-gated Ca^2+^ channels to release glutamate from ribbon-type synapses. ACs and HCs release neurotransmitters from their dendritic trees.

Many questions remain about the distribution and regulation of and the roles played by synaptic proteins at different retinal synapses. For example, certain ACs release both excitatory and inhibitory transmitters, and many questions remain about the specializations that allow differential regulation of different transmitters. The protein complement at many horizontal and AC synapses resembles that of conventional synapses, but AC synapses often vary in their expression of particular isoforms or splice variants. What is the significance of these specific isoforms for shaping responses at these different synapses? The spatial arrangement of proteins at specific synapses and how this arrangement influences function are also poorly understood. In addition to intra- and extracellular scaffolding molecules, there is an emerging understanding of the importance of liquid–liquid phase separation (LLPS) in dynamic regulation of vesicle and protein structures. For example, it is thought that LLPS may help synapsins constrain vesicle mobility (Milovanovic et al. 2018). LLPS might also contribute to the coalescing of Ribeye molecules in a ribbon formation or the clustering of other synaptic proteins. BPCs were one of the early model systems in studying endocytosis, but the sites and mechanisms of endocytosis in other retinal neurons have rarely been investigated. Moreover, we have only begun to unravel the mechanisms for homeostatic regulation of synapses in the retina (Tien et al. 2017). Ongoing work from many laboratories has begun to uncover how these molecules coordinate with one another to give rise to the unique features of synaptic transmission in the retina, but a rich array of questions remain to be answered for a complete understanding of these processes at a molecular level.

## Figures and Tables

**Table 1:** The distribution and putative functions of key presynaptic proteins in the retina

Name	Gene	Differential expression in retinal neurons?	Cell types with high expression	Functions
**SNARE proteins**				
Syntaxin 1a	*STX1a*	No	Amacrine cells, horizontal cells	t-SNARE, exocytosis
Syntaxin 1b	*STX1b*	Yes	Amacrine cells, horizontal cells	t-SNARE, exocytosis
Syntaxin 3	*STX3*	Yes	Rods, cones, bipolar cells, some amacrine cells	t-SNARE, exocytosis
Syntaxin 3b (splice form of *STX3* gene)	*STX3*	Yes	Rods, cones, bipolar cells	t-SNARE, exocytosis
SNAP-25	*SNAP25*	No	High throughout	t-SNARE, exocytosis
Synaptobrevin 1	*VAMP1*	No	Found throughout	v-SNARE, exocytosis
Synaptobrevin 2	*VAMP2*	No	High throughout	v-SNARE, exocytosis
**SNARE accessory proteins**				
MUNC-13–1	*UNC13a*	No	Expressed throughout	Docking, priming
MUNC-13–2	*UNC13b*	Yes	Rods, cones, some amacrine cells	Docking, priming
MUNC-13–3	*UNC13c*	Yes	Type 6 bipolar cells	Docking, priming
MUNC-18a	*STXBP1*	No	High throughout	Priming
MUNC-18b	*STXBP2*	No	High throughout	Priming
MUNC-18c	*STXBP3*	Yes	Rods	Priming
Complexin 1	*CPLX1*	Yes	Amacrine cells, horizontal cells	Exocytosis, fusion clamp
Complexin 2	*CPLX2*	Yes	Horizontal cells, some amacrine cells	Exocytosis, fusion clamp
Complexin 3	*CPLX3*	Yes	Rods, cones, horizontal cells, some amacrine cells	Exocytosis, fusion clamp
Complexin 4	*CPLX4*	Yes	Rods, cones	Exocytosis, fusion clamp
**Active zone proteins**				
Piccolo	*PCLO*	No	Rods, cones, bipolar cells; rarely horizontal and amacrine cells	Active zone scaffold
Piccolino (splice form of *PCLO* gene)	*PCLO*	Yes	Photoreceptors	Active zone scaffold
Bassoon	*BSN*	No	Found throughout	Active zone scaffold
RIM1	*RIMS1*	Yes	Starburst amacrine cells	Active zone, docking?
RIM2	*RIMS2*	Yes	Rods, cones	Active zone, docking?
Ribeye (alternate gene product of *CTBP2*)	*CTBP2*	Yes	Rods, cones, bipolar cells	Ribbon scaffold, docking?, priming?
Rabphilin-3a	*RPH3a*	No	Rod bipolar cells, horizontal cells, starburst amacrine cells	Docking?
**Vesicle proteins**				
Rab-3A	*RAB3A*	No	High throughout	Docking?, priming
SV2a	*SV2A*	No	High throughout	Vesicle associated
SV2b	*SV2B*	Yes	Rods, cones, rod bipolar cells	Vesicle associated
Synaptotagmin 1	*SYT1*	No	High throughout	Calcium triggering of exocytosis, endocytosis, docking?
Synaptotagmin 2	*SYT2*	Yes	Horizontal	Calcium triggering of exocytosis, endocytosis, docking?
Synaptotagmin 4	*SYT4*	Yes	Bipolar cells, horizontal cells, amacrine cells	Calcium triggering of exocytosis, endocytosis, docking?
Synaptotagmin 7	*SYT7*	No	Low expression throughout	Calcium triggering of exocytosis, endocytosis, docking?
Synaptotagmin 9	*SYT9*	Yes	Rod bipolar cells	Calcium triggering of exocytosis, endocytosis, docking?
DOC-2a	*DOC2A*	Yes	Amacrine cells, horizontal cells, bipolar cells	Calcium triggering of exocytosis
**Endocytosis**				
Dynamin 1	*DNM1*	No	High throughout	Vesicle scission
Synaptojanin 1	*SYNJ1*	No	High throughout	Vesicle uncoating
Syndapin	*PACSIN1*	No	High throughout	Vesicle endocytosis
**Calcium channel and associated proteins**				
Ca-α 1A subunit	*CACNA1A*	No	Starburst and A17 amacrine cells	Pore subunit P/Q-type channel
Ca-α 1B subunit	*CACNA1B*	No	Amacrine cells	Pore subunit N-type channel
Ca-α 1D subunit	*CACNA1D*	No	Bipolar cells	Pore subunit L-type channel
Ca-α 1F subunit	*CACNA1F*	Yes	Rods, cones	Pore subunit L-type channel
Ca-α 1H subunit	*CACNA1H*	Yes	AII and A17 amacrine cells, some bipolar cells	Pore subunit T-type channel
Ca-α2δ4 subunit	*CACNA2D4*	Yes	Rods, cones, bipolar cells	Accessory subunit Ca channel
Ca-α2δ3 subunit	*CACNA2D3*	Yes	Rod bipolar cells	Accessory subunit Ca channel
Calcium-binding protein 4	*CABP4*	Yes	Rods, cones	Modulation of calcium channels
Calcium-binding protein 5	*CABP5*	Yes	Bipolar cells, cones	Modulation of calcium channels
**Other proteins**				
Synapsin 1	*SYN1*	Yes	Some amacrine cells, horizontal cells	Vesicle tethering to cytoskeleton
Synapsin 2	*SYN2*	Yes	Some amacrine cells, horizontal cells	Vesicle tethering to cytoskeleton
UNC119	*UNC119*	Yes	Rods, cones, bipolar cells	Lipid-binding chaperone
Rabconnectin 3	*DMXL2*	Yes	Rods, cones, bipolar cells	Docking?

Listed are key presynaptic proteins grouped by their subcellular distribution: SNARE proteins, accessory SNARE proteins, active zone proteins, vesicle proteins, endocytic proteins, proteins associated with Ca^2+^ channels, and other presynaptic proteins. For each, we list the gene name, function, and cell types that express that protein.
